# How Freely Moving Mind Wandering Relates to Creativity: Behavioral and Neural Evidence

**DOI:** 10.3390/brainsci14111122

**Published:** 2024-11-05

**Authors:** Qiuyang Feng, Linman Weng, Li Geng, Jiang Qiu

**Affiliations:** 1 Center for Studies of Education and Psychology of Ethnic Minorities in Southwest China, Southwest University, Chongqing 400715, China; fqiuyang@email.swu.edu.cn; 2 Key Laboratory of Cognition and Personality, Ministry of Education, Southwest University, Chongqing 400715, China; wlm012022306000127@email.swu.edu.cn (L.W.); swugengl@email.swu.edu.cn (L.G.); 3 Faculty of Psychology, Southwest University, Chongqing 400715, China; 4 Collaborative Innovation Center of Assessment toward Basic Education Quality at Beijing Normal University, Southwest University Branch, Chongqing 400715, China

**Keywords:** mind wandering, creativity, neural mechanisms

## Abstract

**Background:** Previous studies have demonstrated that mind wandering during incubation phases enhances post-incubation creative performance. Recent empirical evidence, however, has highlighted a specific form of mind wandering closely related to creativity, termed freely moving mind wandering (FMMW). In this study, we examined the behavioral and neural associations between FMMW and creativity. **Methods:** We initially validated a questionnaire measuring FMMW by comparing its results with those from the Sustained Attention to Response Task (SART). Data were collected from 1316 participants who completed resting-state fMRI scans, the FMMW questionnaire, and creative tasks. Correlation analysis and Bayes factors indicated that FMMW was associated with creative thinking (AUT). To elucidate the neural mechanisms underlying the relationship between FMMW and creativity, Hidden Markov Models (HMM) were employed to analyze the temporal dynamics of the resting-state fMRI data. **Results:** Our findings indicated that brain dynamics associated with FMMW involve integration within multiple networks and between networks (r = −0.11, p_FDR_ < 0.05). The links between brain dynamics associated with FMMW and creativity were mediated by FMMW (c’ = 0.01, [−0.0181, −0.0029]). **Conclusions:** These findings demonstrate the relationship between FMMW and creativity, offering insights into the neural mechanisms underpinning this relationship.

## 1. Introduction

Creativity is defined as the ability of an individual to generate novel and unique products that possess social value [1]. It is associated with various psychological factors, such as emotion [2], self-estimated intelligence [3], and executive function [4]; however, it has not been found to correlate with higher cognitive functioning as measured by neuropsychological tests, including the Digit Span task and the Stroop test [5]. It is regarded as one of the most valuable human qualities, fostering scientific and technological progress as well as societal development. Since the inception of scientific inquiry, narratives have emerged recounting how influential scientists have solved problems during periods of mind wandering (MW). These narratives underscore a universally acknowledged fact: creative activities necessitate that innovators allow their thoughts to wander freely without constraints. Empirical studies complement anecdotal reports by suggesting a relationship between being prone to MW and creative performance in the laboratory [6]. In the study conducted by Baird et al. (2012) [6], participants initially engaged in an unconventional task. They were subsequently randomly assigned to groups performing low-demand tasks, high-demand tasks, resting, or continuous work. The findings indicated that the low-demand group exhibited higher levels of MW during the attentional task and performed better in subsequent divergent thinking tasks following the incubation period. Subsequently, researchers endeavored to replicate Baird et al.’s (2012) [6] study. While some studies have successfully replicated these findings [7], numerous researchers have failed to observe a reliable association between MW and post-incubation creativity [8,9,10]. Due to conflicting outcomes in previous studies, the precise nature of the connection between MW and creativity remains uncertain.

Irving (2022) posits that the ambiguity in the literature may stem from researchers conceptualizing MW as task-unrelated thoughts (TUTs) [7]. However, not all task-unrelated thoughts encompass the form of MW closely linked to creativity, known as freely moving mind wandering (FMMW). This form of MW was initially introduced in a 2016 review article by Christoff, which outlined a dynamic framework of MW [11,12,13]. In this framework, MW is not defined as TUTs [14], but as “thoughts that proceed in a relatively free and unconstrained manner” [11]. The creative thought process encompasses both generation and evaluation [15,16]. The generation phase is characterized by a lack of constraint, while the evaluation phase involves higher levels of constraint. Thought wandering during the incubation period can be especially advantageous for the creative generation phase [12]. This implies that unconstrained thought during incubation should positively correlate with creative generation. Irving et al. (2022) [7] measured thought constraints by incorporating thought probes into the incubation task, asking participants to rate the free movement of their thoughts on a scale of 1–7 [13,17], and found that freely moving thoughts facilitated the generation of creative ideas during moderately engaging activities. This study is the first to demonstrate the relationship between FMMW and creative performance [7].

Over the past decade, integrating neuroimaging with intermittent online experience sampling, where individuals report their thoughts as they occur, has become a well-established and potent technique for correlating brain activity with MW [18,19]. Early studies employing functional MRI (fMRI) in conjunction with these methodologies substantiated the link between MW and the activation of the default mode network (DMN), a finding consistently replicated across various settings [18,19,20]. Subsequent studies have revealed the essential role of widespread, dynamic interactions at the network level. These interactions occur both within the DMN and extend to the frontoparietal control network (FPN) and primary sensory/motor regions, including their functional coupling patterns [21,22]. Research integrating predictive connectome models with fMRI and online experience sampling has demonstrated a reduction in the negative correlation between the DMN and FPN during off-task thought. Another key observation is the reduced connectivity between the DMN and sensorimotor regions, which may facilitate perceptual disengagement [23]. Part of the research focused on individual internal changes in dynamic functional connection (dFC) and its correlation with self-reported MW [24]. For instance, research by Mittner et al. (2014) has linked MW with a range of neural activities, including an upsurge in the DMN, a downturn in the task-positive network, and fluctuations in neuromodulation [25]. Similarly, Kucyi and Davis (2014) discovered that an overall heightened state of MW may indicate more fluid interactions within the DMN territories [26]. Crucially, this research suggests that dFC assessments might outperform static ones in forecasting MW variability on an individual basis [26]. Collectively, these insights highlight the dynamic states of within- and between-network functional connectivity (FC) of the DMN during MW.

Previous research on creativity has predominantly utilized divergent thinking task paradigms, such as the Alternate Uses Task (AUT) [27], creative storytelling [28], and metaphor generation [29]. The AUT is the most widely employed and thoroughly validated paradigm for assessing creative thinking. In this experimental task, participants are presented with the names of everyday objects and are asked to think of novel and unusual uses for these items. Beaty and colleagues integrated the AUT with fMRI to analyze the FC data of participants and replicated the analysis in three independent samples [30]. The results indicated that the FC characteristics of the DMN, FPN, and salience network (SN) could effectively predict individual differences in creative thinking [30].

Previous studies have used the Sustained Attention To Response Task (SART) to measure FMMW, we first assessed the construct validity of the questionnaire for measuring FMMW by examining the relationship between FMMW as measured by the questionnaire and during the SART and by comparing their associations with further variables that earlier studies described to be associated with FMMW (positive affect [31]). Then, we examined the relationships between FMMW, creative performance, and their underlying neural mechanisms. Given the dynamic nature of FMMW, we employed dynamic magnetic resonance analysis techniques—hidden Markov models (HMM). In the exploration of brain dynamics at rest, HMM is an effective method for detecting rapid processes that may not be captured by other analytical techniques [32]. It was hypothesized that individual creative performance would be linked to FMMW. FMMW might be reflected in the dynamic activities of the brain. Finally, the mediation analysis revealed that FMMW played a mediating role between its related brain dynamic and creativity.

## 2. Methods

### 2.1. Participants

The study comprised two samples. Sample 1 consisted of 163 participants, including 49 men and 114 women aged between 18 and 26 years (mean age = 21.14, SD = 1.69). They completed the FMMW questionnaire, Sustained Attention to Response Task (SART), and Positive and Negative Affect Scale (PANAS). Sample 2 was obtained from a large database established by the Behavioral Brain Research Project of Chinese Personality (BBP). This project collected a range of psychological variables, including creativity and emotional well-being, aiming to elucidate the neural and genetic bases of mental health and complex cognitive functions. Participants were recruited from Southwest University by means of the campus network, advertisements on bulletin boards and leaflets, or through face-to-face communications on campus. Prior to participating in the project, they were required to pass a series of experimental screenings, including assessments of psychiatric history, mental health status, handedness, and MRI contraindications. All participants were required to be healthy and right-handed, with no history of psychiatric disorders, substance abuse (including illegal drugs and alcohol), or any contraindications for MRI. The project leader explained the details of the experiment to the participants, and upon agreeing to participate, each signed informed consent forms. This study was approved by the Ethics Committee of the Brain Imaging Center of Southwest University. A total of 1424 participants from the BBP completed the assessments, including the Freely Moving Thought scales, creative tasks, and resting-state scan. To ensure the accuracy and reliability of the study results, we excluded “outliers”, defined as participants with z-scores exceeding ± 3 [33]. Additionally, to minimize the impact of motion artifacts on the functional connectivity analysis, we excluded participants who exhibited excessive head motion during resting-state scans, defined as those with an average framewise displacement (FD) greater than 0.3 [34]. The final analysis included 1316 participants, comprising 427 men and 889 women, aged between 18 and 26 years (mean age = 18.89, SD = 0.92).

### 2.2. Behavioral Data

#### 2.2.1. Freely Moving Mind Wandering Scale (FMMW-S)

We assessed the level of participants’ FMMW using the item “My mind seems to be pulled from one subject to the next when my mind is wandering” from the Intentional and Unintentional Mind Wandering Scale developed by Carriere et al. (2013) [35]. Participants rated aspects of their everyday experiences of MW on a 7-point Likert scale (1 = rarely, 7 = frequently).

#### 2.2.2. Sustained Attention to Response Task (SART)

Previous studies have used the SART to measure FMMW. To verify the validity of our questionnaire measurement, the SART was employed to assess the level of FMMW and explore its correlation with the questionnaire results.

**Figure 1:**
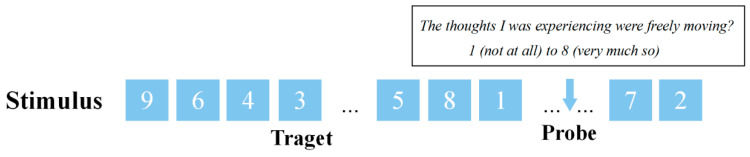
An example of the SART process.

The SART is a Go/No Go paradigm [36], in which digits ranging from 1 to 9 are randomly displayed on a computer screen. Participants are required to press a key in response to any digit except 3 (Go stimuli: 1, 2, 4–9), while no response is needed when the digit 3 (No Go stimulus) appears (see Figure 1). During the experiment, thought probes were randomly presented to assess the level of free flow in participants’ current thoughts. The SART consists of 30 No Go trials, 222 Go trials, and 3 thought probes arranged in a pseudo-random order. Notably, No Go trials do not occur consecutively nor precede thought probes. At the commencement of the experiment, a fixation cross is presented at the center of the screen for 1250 ms, followed by a stimulus presented for 1250 ms. Prior to the main experiment, participants undergo practice trials comprising 17 Go trials, 1 No Go trial, and 1 thought probe. The questions for the thought probes are determined based on previous research on MW [7,9]. Upon the appearance of a thought probe, the task is paused, and participants are asked to rate the statement: “The thoughts I was experiencing were freely moving”, using an 8-point Likert scale. The score for FMMW is calculated as the average of the responses to the three probes answered by the participant.

#### 2.2.3. Positive and Negative Affect Scale (PANAS)

The PANAS, developed by Watson and colleagues in 1988 [37], is a self-report questionnaire consisting of 10 items to assess positive affect and another 10 items to measure negative affect. Each item is scored on a 5-point scale from 1 (not at all) to 5 (very much). In this research, we concentrated exclusively on the total positive affect score, termed PANAS_PA_Score.

#### 2.2.4. Alternative Uses Task (AUT)

During the implementation of the BBP, participants engaged in an Alternative Uses Task (AUT) to measure creative thinking [38]. In this task, they were given 3 min to generate as many unique and appropriate uses of bricks and cans as possible. Each item was displayed individually on the screen. Upon formulating an idea, participants pressed the “1” key and typed it out. This procedure continued until the 3 min duration was complete. The task was presented using E-Prime 2.0 software.

In alignment with methods from earlier studies, AUT responses were assessed by four trained raters. The assessment included all responses for both objects, focusing on four dimensions of creativity: fluency (number of ideas generated per object), flexibility (number of categories generated per object), originality (uniqueness on a 5-point scale from 1 (least original) to 5 (most original)), and appropriateness (suitability on a 5-point scale from 1 (very unsuitable) to 5 (highly suitable)). The inter-rater reliability analysis showed high agreement among raters, with coefficients ranging from 0.728 to 0.984.

In this study, appropriateness was excluded from the analysis as it was unrelated to the research question. The dimensions of fluency, flexibility, and originality were included in subsequent analyses. Ratings for the three dimensions provided by the four assessors for both items (cans and bricks) were averaged. Subsequently, the average scores for each dimension across the two items were computed. To derive overall creativity performance for each task, we computed the total z-scores across the three dimensions for subsequent analysis.

#### 2.2.5. The Inventory of Creative Activities and Achievements (ICAA)

We used the ICAA to assess participants’ engagement in real-life creative activities [39]. The questionnaire provided scores reflecting the frequency of involvement in various creative domains (arts and crafts, literature, creative cooking, music, sports, performing arts, science and engineering, and visual arts). Each domain included six items that assessed the frequency of activities over the past 10 years. Responses were recorded on a 5-point Likert scale: 0 (never), 1 (1–2 times), 2 (3–5 times), 3 (6–10 times), and 4 (more than 10 times). The ICAA score was calculated by summing the ratings across all eight domains.

### 2.3. Image Acquisition and Preprocessing

All functional and structural data were obtained using a 3T SIEMENS scanner (Erlangen, Germany) at the Brain Imaging Center of Southwest University. The specific scanning parameters are detailed in our previously published paper [27].

Data Processing and Analysis for (Resting-State) Brain Imaging [40], based on statistical parametric mapping software (SPM12, https://www.fil.ion.ucl.ac.uk/spm/software/spm12/, accessed on 1 May 2024), were used to preprocess fMRI data. The preprocessing steps were carried out as follows: The original DICOM format data were converted to the NIFTI format; the first 10 time points were excluded to eliminate the impact of the participants’ initial adaptation to the scanner and the instability of the MRI signal on the data. The remaining 232 time-point images were subjected to temporal slice correction, head motion correction, and registration to the individual’s T1 structural image, followed by spatial normalization with a voxel size of 3 × 3 × 3 mm. Spatial smoothing was applied to the images using a Gaussian kernel with a full width at half maximum of 4 mm, and linear trends were removed. Nuisance signals, such as white matter, cerebrospinal fluid, and head-motion parameters, along with their derivatives, were regressed using the Friston 24-parameter model. Finally, filtering (0.01–0.08 Hz) was performed.

### 2.4. Resting-State Time Series Extraction

We employed the Schaefer 400 Parcels (17 networks) Atlas [41], a recently developed cortical parcellation based on fMRI data, to delineate cortical regions of interest. This atlas partitions the cortex into 17 distinct functional networks through network-specific parcellations. To generate a time series for the entire brain, we employed the Graph Theoretical Network Analysis toolbox to calculate the time course of each node [42]. This process entailed averaging the BOLD signals across all voxels within each node at every time point. Subsequently, the obtained time series data were standardized and concatenated to create a matrix with dimensions of (1316 participants × 235 volumes) × 17 (representing the average signal extracted from the 17 network masks). This matrix served as the input for the HMM.

### 2.5. Hidden Markov Model (HMM)

To capture the dynamics of neural activity, we employed the HMM, which is a framework that assumes the presence of distinct states recurring intermittently over time [43,44]. The estimation of model parameters followed a variational Bayes approach and involved minimizing free energy using the HMMMAR toolbox [32] available at https://github.com/OHBA-analysis/HMM-MAR, accessed on 1 May 2024

The Hidden Markov Model (HMM) output reveals the predominant brain state at each time point, showcasing the temporal dynamics during resting state using two critical statistics: lifetime and state transition matrices. Lifetime refers to the average duration that each participant spends in a specific state before transitioning to another state. For each participant, a state transition matrix is constructed, which represents the probabilities of transitioning from one state to another. To compute these statistics, we employed Viterbi decoding, which involves a hard classification of states as active or inactive at each time point based on the most likely path through the states. Subsequently, the lifetime and state transition matrices were calculated based on this classification, offering quantitative measures of the temporal dynamics underlying the R-fMRI data.

### 2.6. Mediation Analysis

To investigate the potential collaborative influence of FMMW and associated brain patterns on creativity, mediation analyses were conducted, focusing on the mediating role of FMMW between neural networks and creativity. In the mediation model, FMMW-related brain patterns were designated as independent variables, with creativity scores as dependent variables. FMMW was used as the mediating variable. Mediation analyses were performed using the PROCESS macro [45] in SPSS 24.0 statistical software. The indirect effect was determined by multiplying the regression coefficient (path a) between brain patterns and FMMW with the regression coefficient (path b) between FMMW and creativity. To assess the significance of the indirect effect, we employed a bootstrapping method, specifically conducting 1000 bootstrap resampling iterations, allowing for the estimation of the confidence interval to determine statistical significance.

## 3. Results

### 3.1. Descriptive Analyses

Table 1 and Table 2 present descriptive statistics for all measures in Sample 1 and Sample 2, respectively. The tables show that the skewness and kurtosis values for all variables fall within acceptable limits (skewness < 2, kurtosis < 4), suggesting that the distributions are normal for these measurements. Pearson product-moment correlations were used to calculate the correlations.

### 3.2. Construct Validity

To investigate the construct validity of FMMW-S, we examined the relationship between the FMMW-S, FMMW-SART, and PA. Pearson correlation analysis revealed a positive correlation between FMMW-S and FMMW-SART (r = 0.30, *p* < 0.001) in Table 3. Table 3 shows a positive correlation between FMMW-S and PA (r = 0.21, *p* = 0.008), as well as a positive correlation between FMMW-SART and PA (r = 0.24, *p* = 0.002). This indicates that the two measures of FMMW had similar correlations with PA. The results indicated a correspondence between FMMW measurements obtained via the two methods. This correspondence confirmed the validity of the questionnaire as a measure of FMMW.

### 3.3. Relationship Between FMMW and Creativity

The Pearson correlation coefficients for all measures in Sample 2 are presented in Table 4. To support the results of the correlation analysis, we computed Bayes factors for the correlations. We observed a significant positive correlation between FMMW and the fluency dimension of the AUT, r = 0.10, *p* < 0.001, with strong evidence in favor of the positive association over the null BF_+0_ = 41.88. FMMW was also positively associated with the flexibility dimension of AUT responses, r = 0.09, *p* = 0.001, with strong evidence in favor of the positive association over the null BF_+0_ = 21.71. Likewise, FMMW was positively associated with the originality dimension of AUT responses, r = 0.10, *p* < 0.001, with strong evidence in favor of the positive association over the null BF_+0_ = 34.00. We found a small, significant, correlation between FMMW and ICAA scores, r = 0.07, *p* = 0.012. However, there was no strong evidence for the positive association over the null, BF_+0_ = 1.56. The results showed significant positive associations between the FMMW and creative thinking (AUT).

### 3.4. Brain State Lifetime Is Associated with Dynamic Features of FMMW

**Figure 2:**
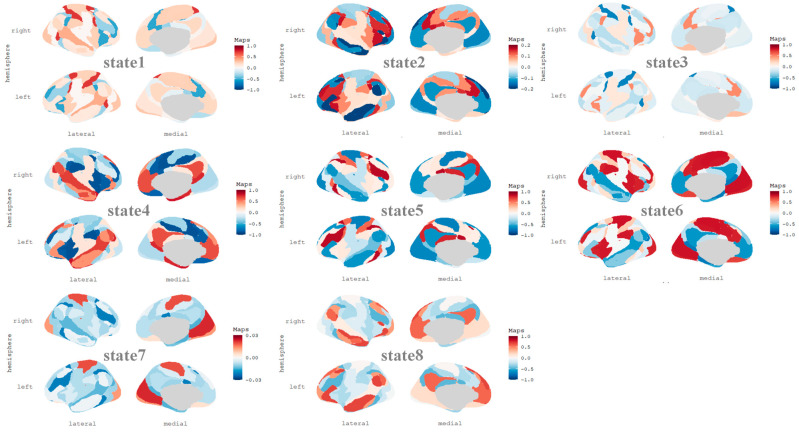
The functional magnetic resonance imaging signal profiles corresponding to each brain state detected during the scan, as identified by the hidden Markov model. The panels display the spatial distribution of the average activation within each state, represented by a blue-red color bar that signifies the relative loading with respect to the mean activation.

**Figure 3:**
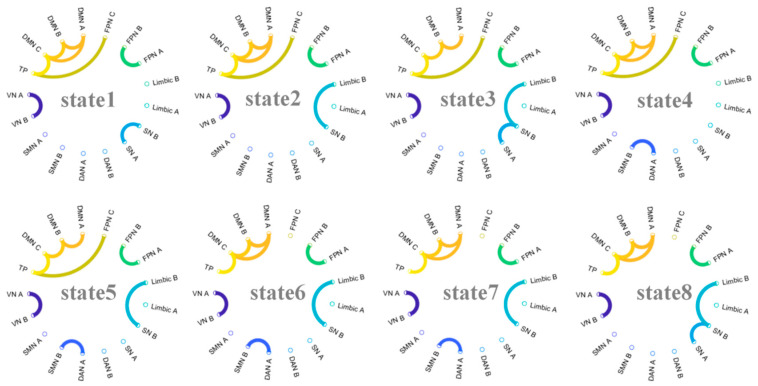
The functional magnetic resonance imaging signal profiles corresponding to each brain state detected during the scan, as identified by the hidden Markov model. In the panels, the top 5% of positive functional connectivity is depicted, with distinct colors denoting each of the 17 subnetworks.

We successfully identified and characterized eight HMM states in our study. The fMRI signal profiles for each state are depicted in Figure 2 and Figure 3. Figure 2 illustrates the spatial distribution of average brain activation across states 1 to 8, while Figure 3 presents the top 5% of functional connectivity strength among the 17 functional networks. To explore the relationships between brain states and FMMW, we calculated the lifetime of the eight states across all participants. Notably, our findings revealed that only state 6 exhibited a statistically significant positive association with FMMW (r = −0.11, p_FDR_ < 0.05). After controlling for age and gender, state 6 was positively correlated with the FMMW (r = −0.11, p_FDR_ < 0.05).

State 6 demonstrated a prominent positive activation pattern within several key neural networks, including the visual network (VN), salience network (SN), subsystem of somatomotor network (SMN), and limbic system (Figure 2). The FC values within DMN (r = 0.72 to 0.79), FPN (r = 0.74), VN (r = 0.75), and between DANA and SMNB (r = 0.72), as well as SNB and LimbicB (r = 0.71), were positively correlated (Figure 3). Additionally, we observed a weak positive correlation between the FC of the DMN and the FPN (r = 0.17 to 0.52).

### 3.5. Mediation Analysis

**Figure 4:**
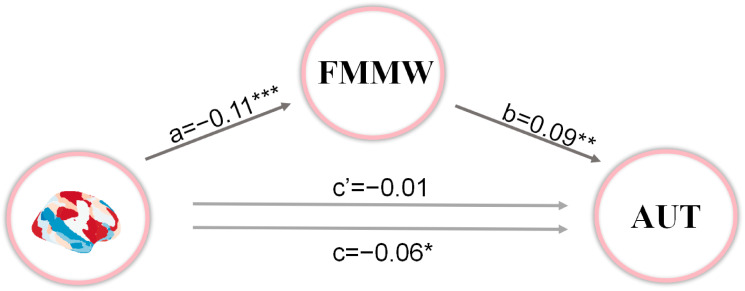
Mediation analysis. The mediating role of FMMW on the relationship between brain patterns and AUT sum score. FMMW, freely moving mind wandering; AUT, the Alternative Uses Task; * *p* < 0.05; ** *p* < 0.01; *** *p* < 0.001.

As shown in Figure 4, we constructed a mediation model, which reveals a statistically significant relationship between brain patterns and FMMW (a = −0.11, *p* < 0.001), as well as between FMMW and the AUT sum score (b = 0.09, *p* = 0.001). The calculated indirect effect, using bootstrapped estimates, was −0.01 with a 95% confidence interval of −0.0181 to −0.0029, indicating statistical significance. Conversely, the direct effects did not reach statistical significance (β = −0.05, *p* = 0.086). Therefore, FMMW mediates the relationship between brain dynamics and AUT scores.

## 4. Discussion

In this study, we first demonstrated the validity of the questionnaire for measuring FMMW. We collected data from 1316 participants to examine the relationship between FMMW and creative performance. Correlation analysis and Bayes factors revealed that individual creative thinking (AUT) was associated with FMMW. To elucidate the neural mechanisms underlying the relationship between FMMW and creativity, HMM was used to calculate the temporal dynamics of the R-fMRI data. Notably, our findings indicated that brain dynamics related to FMMW were reflected in integration within and between multiple networks. The links between lifetime on the related brain state and creativity were mediated by FMMW. These findings demonstrated the relationship between FMMW and creativity and explained the neural mechanisms underlying this relationship by probing the brain dynamics and mediating analysis.

First, in this study, we revealed that FMMW was positively correlated with the three dimensions of creative thinking (AUT) and creative behavior (ICAA). Bayes factors provided strong evidence supporting the positive correlation between FMMW and the three dimensions of creative thinking (BF_+0_ > 10, indicating significant support for the alternative hypothesis while rejecting the null hypothesis). However, it did not provide supporting evidence for the positive correlation between FMMW and creative behavior (BF_+0_ = 1.56, indicating similar support for both the null and alternative hypotheses and hence failing to reject the null hypothesis) [46]. In summary, FMMW shows a significant positive correlation with creative thinking. Previous research found that FMMW during moderately engaging incubation activities facilitated the generation of creative ideas [7]. Our study employed a large sample and found that the relationship between FMMW and creativity exists not only during the incubation phase but also that individuals with more FMMW in their daily lives are inherently more creative. The dynamic theory of MW provides a potential explanation [11,47]. Based on this theory, both MW and creativity are types of spontaneous thought within the same cognitive process category, involving a wide-ranging, associative, and exploratory thinking style [7,11,12]. FMMW is lightly constrained by the control and salience networks, which allows it to drift widely among loosely connected ideas, offering particular advantages during the generation phase of creative thinking [12].

To explore the neural mechanisms underlying FMMW, we employed the HMM on fMRI data, a widely used machine learning method for capturing brain dynamics [44,48]. We found that participants with higher FMMW exhibited shorter lifetimes in the unique state associated with positive activation of the VN, SMN, SN, and Limbic networks. Additionally, there were interactions within FPN, DMN, and VN, and between DANA–SMNB, SNB–LimbicB, and FPN-DMN connectivity. The DAN is considered to aid in focused attention on sensory aspects of the environment and to associate this sensory information with motor responses. It is primarily engaged when we direct our focus to the external surroundings [49]. The SN may serve to instinctively guide or redirect attention to salient perceptual stimuli, both external and internal, especially when attention is captured in a “bottom-up” manner [50]. Interactions between the DAN and SMN, SNB, and LimbicB might support automatic constraints on thought, thereby constraining the spontaneous movement of attention. The FPN is engaged in goal-directed cognition that encompasses both internal and external orientations. The FPN can engage in positive functional connectivity with the DMN to facilitate intentional and inward-focused autobiographical planning. The interactions within FPN and DMN might support deliberate constraints on thought. In conclusion, interactions between the SN and DAN and other networks support automatic constraints on the thought, and interactions between FPN and DMN impose conscious constraints on the thought, acting together to limit the free flow of the thought.

Finally, our findings indicate that FMMW mediates the relationship between brain dynamics and creativity. Previous studies have demonstrated that individuals with high creative thinking abilities exhibit consistent patterns of functional brain connectivity, including FPN, SN, and DMN [30]. According to a recent brain network framework based on the dual-process theory of creativity [51], the DMN facilitates idea generation, while the FPN aids in idea assessment, aligning with recognized functions in mental simulation and executive cognition. Moreover, the SN, essential for detecting behaviorally significant cues and enabling dynamic transitions between default and executive systems, can identify potential ideas originating from creative processes within the DMN and relay such insights to executive networks for a more sophisticated analysis [16]. State 6 is characterized by strong connectivity within the DMN and FPN, with weaker coupling between the two networks. This diminished interaction may be detrimental to the generation of creative ideas. Our mediation analysis suggested that the shorter the duration of this brain dynamic pattern, the more spontaneous thought flows, leading to higher levels of creativity.

Nevertheless, this study has some limitations. First, the dynamic framework of mind wandering is a relatively recent concept, introduced only a few years ago. Consequently, there is a limited amount of existing research in this area, which restricts the availability of variables that can be used to validate the construct validity of the FMMW-S. Secondly, we explored the brain dynamics of FMMW at the trait level. Future research should assess the brain dynamics of FMMW at the state level. Thirdly, our study only explored the neural mechanisms underlying the relationship between FMMW and creativity, and future research could employ an incubation effect experimental paradigm to explore the neural mechanisms of FMMW during incubation tasks and its effect on creative performance post-incubation. Finally, one significant aspect that warrants attention is the absence of an exploration of cross-cultural influences on the research findings. While our investigation provides valuable insights into the relationship between FMMW and creativity, the lack of a cultural context may restrict the generalizability of our results.

## 5. Conclusions

This study highlights that FMMW is positively correlated with creative thinking (AUT). At the neural level, we applied the HMM to resting-state fMRI data, and found that participants with higher FMMW exhibited shorter lifetime in a unique state associated with positive activation of the VN, SMN, SN, and Limbic networks, along with interactions within FPN, DMN, and VN, as well as the DANA–SMNB, SNB–LimbicB connectivity. Furthermore, mediation analysis revealed that FMMW mediates the relationship between brain dynamics and creativity. In summary, this study provides a deeper understanding of the relationship between MW and creativity within a dynamic framework. In the future, it will be possible to further explore how FMMW during incubation tasks influences the incubation of creativity. Our research offers valuable insights into the enhancement of individual creativity. Specifically, it suggests that fostering the level of FMMW may serve as an effective strategy for augmenting creative potential.

## Figures and Tables

**Table 1:** Descriptive statistics in Sample 1.

	M	SD	Range	Skewness	Kurtosis
FMMW-S	4.96	1.38	2.00–7.00	−0.46	−0.53
FMMW-SART	4.78	1.67	1.00–8.00	0.13	−0.69
PANAS_PA	28.27	7.79	10–45	−0.28	−0.20

**Table 2:** Descriptive statistics in Sample 2.

	M	SD	Range	Skewness	Kurtosis
FMMW-S	4.95	1.22	2.00–7.00	−0.37	0
AUT_Flu	6.00	2.16	1.40–12.70	0.55	−0.02
AUT_Fle	4.83	1.53	1.00–9.83	0.37	−0.09
AUT_N	15.41	6.20	2.00–35.67	0.62	0.07
ICAA	29.89	19.05	0–93.00	0.74	0.11

**Table 3:** The correlations among the study variables in Sample 1.

	1	2	3
FMMW-S	-		
FMMW-SART	0.30 ***	-	
PANAS_PA	0.21 **	0.24 **	-

*** *p* < 0.001, ** *p* < 0.01.

**Table 4:** The correlations among the study variables in Sample 2.

	1	2	3	4	5
FMMW-S	-				
AUT_Flu	0.10 ***	-			
AUT_Fle	0.09 **	0.96 ***	-		
AUT_N	0.10 ***	0.96 ***	0.95 **	-	
ICAA	0.07 *	0.24 ***	0.24 **	0.24 **	-

* *p* < 0.05, ** *p* < 0.01, *** *p* < 0.001.

## Data Availability

The data and material used in this study are available from the corresponding author upon reasonable request.
